# 2D Ruddlesden-Popper Perovskite (C_6_H_5_NH_3_)_2_CsPb_2_Cl_7_ with Favorable Radiative Recombination and Field-Effect Transport

**DOI:** 10.3390/ma19101991

**Published:** 2026-05-11

**Authors:** Zhe Pang, Yuxuan Wang, Chong Peng, Yingfei Liu, Jiaqian Que, Kefeiyang Hu, Xingbo Huang, Yong Liu

**Affiliations:** 1 State Key Laboratory of Advanced Technology for Materials Synthesis and Processing, School of Materials Science and Engineering, Wuhan University of Technology, Wuhan 430070, China; 15091422674@163.com (Z.P.); 15856589663@163.com (C.P.); liu714961@163.com (Y.L.); 294599@whut.edu.cn (J.Q.); hkfy8789@whut.edu.cn (K.H.); 15251300417@163.com (X.H.); 2 School of Mechanical and Electrical Engineering, Wuhan University of Technology, Wuhan 430070, China; 19870978239@163.com

**Keywords:** 2D RP perovskites, radiative and non-radiative recombination rates, photophysical properties, carrier mobility

## Abstract

Organic–inorganic hybrid halide perovskites have attracted extensive attention due to their excellent optoelectronic properties and potential applications in field-effect transistors (FET), light-emitting diodes (LEDs), and photodetectors. However, conventional three-dimensional (3D) perovskites are limited by intrinsic instability and ion migration. Two-dimensional Ruddlesden-Popper (2D RP) perovskites offer improved structural stability, but many systems still suffer from modest photoluminescence efficiency and limited charge-transport performance. In this work, a novel 2D RP perovskite, (C_6_H_5_NH_3_)_2_CsPb_2_Cl_7_, was designed and synthesized, where the anilinium ion (C_6_H_5_NH_3_^+^) serves as the organic spacer. Structural characterization indicates that the material possesses high crystallinity and a smooth surface morphology. Optical measurements reveal a violet emission peak at 411 nm with a single-peak feature and a full width at half maximum (FWHM) of 10 nm. The bandgap is determined to be 3.1 eV. Time-resolved photoluminescence (TRPL) measurements show an average lifetime of 4 ns, and the photoluminescence quantum yield (PLQY) is 29.8%. Based on the measured PLQY and lifetime, the radiative and non-radiative recombination rates were estimated to be K_r_ ≈ 7.45 × 10^7^ s^−1^ and K_nr_ ≈ 1.76 × 10^8^ s^−1^, respectively, suggesting that radiative recombination is appreciable although non-radiative pathways remain present. FET measurements demonstrate an on/off current ratio of 10^4^ and a carrier mobility of 1.1 cm^2^ V^−1^ s^−1^. Without any systematic optimization, (C_6_H_5_NH_3_)_2_CsPb_2_Cl_7_ exhibits relatively favorable emissive behavior and measurable field-effect charge transport performance when compared with structurally similar 2D RP perovskites reported under comparable, non-optimized conditions. This study expands the family of chloride-based 2D perovskites and provides a basis for future improvements in their recombination and field-effect transport properties.

## 1. Introduction

Organic–inorganic hybrid halide perovskites have attracted extensive attention in recent years owing to their high absorption coefficients, tunable bandgaps, and favorable charge-transport properties, and have been widely investigated for applications in field-effect transistors (FET), light-emitting diodes (LED), and photodetectors [1,2,3]. However, conventional three-dimensional (3D) perovskites still suffer from insufficient stability in practical applications. Under external stresses such as humidity, heat, and continuous illumination, their structures are prone to degradation. Meanwhile, ion migration and nonradiative recombination induced by grain-boundary defects further deteriorate device performance and long-term operational stability [4,5,6].

To mitigate the limitations of 3D perovskites in terms of stability and ion migration, 2D RP perovskites have attracted increasing attention. Their general chemical formula is L_2_A_n−1_B_n_X_3n+1_, where L represents bulky organic spacer cations (e.g., BA^+^, PEA^+^), A denotes small cations (e.g., Cs^+^, MA^+^, FA^+^), B corresponds to metal cations (e.g., Pb^2+^, Sn^2+^, Cd^2+^), and X stands for halide anions (Cl^−^, Br^−^, I^−^). In contrast to 3D perovskites, 2D RP perovskites consist of alternately stacked inorganic octahedral layers and organic spacer layers, forming a natural multiple-quantum-well (MQW) structure. This layered configuration spatially confines charge carriers within the inorganic slabs, while the organic spacers act as potential barriers, which not only enhance structural stability but also partially hinder ion migration pathways, thereby suppressing ion migration to some extent [7,8,9]. In addition, the quantum confinement induced by the two-dimensional layered structure, together with the pronounced dielectric mismatch between the organic and inorganic components, further strengthens dielectric confinement and thereby increases the exciton binding energy. A higher exciton binding energy is beneficial for stabilizing excitonic states and may facilitate radiative recombination under certain conditions [10,11,12,13]. At the same time, however, stronger carrier confinement may suppress the generation and transport of free carriers, leading to an intrinsic trade-off between luminescence efficiency and charge-transport performance. It should be noted that the magnitude and physical significance of the exciton binding energy in halide perovskites remain under debate. This value is not a fixed constant, but is closely related to the material system, measurement method, and analytical model employed. Previous studies have shown that the exciton binding energies of two-dimensional perovskites are generally much higher than those of three-dimensional perovskites, typically ranging from tens to hundreds of meV [14,15,16]. Despite the advantages of RP perovskites in terms of structural stability and suppressed ion migration, achieving a balance between efficient radiative recombination and high field-effect charge transport remains challenging [17,18,19]. Without systematic optimization (e.g., doping or compositional engineering), RP systems often struggle to simultaneously realize high radiative efficiency and efficient carrier transport [20,21,22,23,24]. This issue is more pronounced in chloride-based systems, where systematic investigations into their optoelectronic properties remain relatively limited compared to their bromide and iodide counterparts.

To address the challenges, we designed and synthesized a novel layered chloride RP perovskite, (C_6_H_5_NH_3_)_2_CsPb_2_Cl_7_, in which the C_6_H_5_NH_3_^+^ (anilinium ion) serves as the organic spacer. Structural and morphological characterizations indicate that the material exhibits high crystallinity with a relatively smooth surface. Optical measurements reveal violet emission at 411 nm with a narrow spectral width (FWHM ≈ 10 nm). Ultraviolet photoelectron spectroscopy (UPS) and Ultraviolet–visible spectroscopy (UV–Vis) measurements were combined to construct the band alignment, giving a bandgap of 3.1 eV and a Fermi level slightly closer to the conduction band, consistent with weak n-type behavior. TRPL measurements yield an average carrier lifetime of 4 ns, and the photoluminescence PLQY is 29.8%. Based on the measured PLQY and carrier lifetime, the radiative and non-radiative recombination rates are estimated to be on the order of 10^7^ and 10^8^ s^−1^, indicating that radiative recombination contributes significantly to the overall decay process, although non-radiative pathways are still present. FET measurements show that the device exhibits an on/off current ratio of ~10^4^ and a carrier mobility up to 1.1 cm^2^ V^−1^ s^−1^. Compared with structurally related 2D RP perovskites reported under similar non-optimized conditions, (C_6_H_5_NH_3_)_2_CsPb_2_Cl_7_ shows a relatively favorable combination of radiative recombination and field-effect transport characteristics. These results expand the family of chloride-based 2D RP perovskites and provide a useful basis for further studies on composition modulation, structure-property relationships, and device optimization.

## 2. Materials and Methods

### 2.1. Materials

Precursor: Hydrochloric acid aniline (C_6_H_5_NH_2_HCl) (99%), Cesium chloride (CsCl) and (99%), Lead chloride (PbCl_2_) (99%) were purchased from Macklin (Shanghai, China), and hydrochloric acid (HCl) (36 wt%) was obtained from Sinopharm Group (Beijing, China); Gas: Nitrogen (N_2_ gas, Wuhan Newradar Special Gas Co., Ltd., 99.999%, Wuhan, China); Substrate: Si/SiO_2_ (Hefei Kejing Materials Technology Co., Ltd., Hefei, China).

### 2.2. Methods

The 2D layered perovskite (C_6_H_5_NH_3_)_2_CsPb_2_Cl_7_ was fabricated through a cooling crystallization protocol under a continuous nitrogen (N_2_) purge atmosphere to avoid moisture interference. Specifically, C_6_H_5_NH_2_HCl (81.6 mg, 0.63 mmol), CsCl (168.4 mg, 1 mmol), and PbCl_2_ (347.6 mg, 1.25 mmol) were dissolved in concentrated hydrochloric acid in strict accordance with the predesigned stoichiometric ratio. The resulting homogeneous solution was first heated to 120 °C to ensure full dissolution of all precursors, followed by natural cooling to 50 °C and a subsequent reheating step to 70 °C. Subsequently, the solution was slowly cooled to room temperature, and the supernatant was separated and collected to obtain the target product (C_6_H_5_NH_3_)_2_CsPb_2_Cl_7_.

### 2.3. Characterization

#### 2.3.1. Characterization of Material Morphology

Microscopic morphology was characterized by a Hitachi S-4800 field emission scanning electron microscope (SEM, Hitachi S-4800, Hitachi, Tokyo, Japan) and elemental distribution content analysis by energy dispersive X-ray spectroscopy (EDS) (Bruker Nano GmbH, Berlin, Germany).

#### 2.3.2. Structural and Surface Characterizations

X-ray diffraction (XRD), X-ray photoelectron spectroscopy (XPS), and Fourier transform infrared (FTIR) spectroscopy were performed to characterize the crystal structure, chemical states, and functional groups of the as-prepared samples, respectively. XRD measurements were carried out on a Bruker D8 X-ray diffractometer equipped with a Cu Kα radiation source (λ = 1.54056 Å) under an operating voltage of 40 kV and an operating current of 40 mA (Bruker AXS, Karlsruhe, Germany), where the test sample was prepared by drop-casting a diluted hexane suspension of (C_6_H_5_NH_3_)_2_CsPb_2_Cl_7_ perovskite onto a SiO_2_/Si substrate, followed by natural drying. XPS analysis was conducted on a K-Alpha XPS spectrometer (Thermo Fisher Scientific, Waltham, MA, USA) with a monochromatic Al Kα X-ray source, and the binding energy scale was calibrated with the C 1s peak at 284.8 eV corresponding to the C-C bond. FTIR spectra were recorded on a Nicolet 6700 FTIR spectrometer (Waltham, MA, USA), for which the analyte sample was fabricated by drop-casting the concentrated nanorod solution onto a clean glass substrate.

#### 2.3.3. Optical Characterization of Materials

UV–Vis absorption, steady-state photoluminescence (PL), and time-resolved photoluminescence (TRPL) measurements were conducted to characterize the optical properties of the as-prepared samples. UV–Vis absorption spectra were acquired on a Shimadzu UV-1800 spectrophotometer (Kyoto, Japan) using the supporting UVProbe software (version 2.52). Steady-state PL spectra were recorded on a Shimadzu RF-6000 spectrophotometer (Shimadzu Corporation, Kyoto, Japan) with LabSolutions RF software (version 5.82) at an excitation wavelength (λ_ex_) of 325 nm. TRPL spectra were measured at the emission wavelength of 411 nm on a TRPL spectrometer (Nano LED-C2 N-485L, Horiba Scientific, Kyoto, Japan), with a 295 nm picosecond pulsed diode laser (pulse width: ~200 ps, pulse energy: 14 pJ) as the excitation source. The PLQY was measured using an Edinburgh FLS-1000 fluorescence spectrometer (Edinburgh Instruments, Livingston, UK), equipped with the corresponding data acquisition software. The excitation wavelength (λ_ex_) was set at 280 nm, with a scanning range of 250–600 nm.

#### 2.3.4. Fabrication and Electrical Characterization of Material-Based Field-Effect Transistors

The fabrication and electrical characterization of the perovskite field-effect transistors (FET) were conducted as follows. A customized shadow mask was fixed onto the silicon substrate supported with the as-prepared 2D perovskite using an ET-1 2D material transfer platform (Mitsui Optoelectronics Co., Ltd., Kitakyushu, Japan). Silver (Ag) was precisely deposited on both ends of the perovskite as the source and drain (S/D) electrodes via thermal evaporation using a PD400S high vacuum evaporation coating system (Wuhan Pudi Vacuum Technology Co., Ltd., Wuhan, China). The electrical characteristics of the as-fabricated FET were measured and analyzed using a B1500A semiconductor (Keysight Technologies, Inc., Singapore).

## 3. Results

This study employed a cooling crystallization method to synthesize 2D RP phase perovskite (C_6_H_5_NH_3_)_2_CsPb_2_Cl_7_. Specifically, aniline hydrochloride (C_6_H_5_NH_2_·HCl, 81.6 mg, 0.63 mmol), CsCl (168.4 mg, 1 mmol), and PbCl_2_ (347.6 mg, 1.25 mmol) were dissolved in concentrated hydrochloric acid according to the designed stoichiometric ratio. In the concentrated hydrochloric acid medium, C_6_H_5_NH_2_·HCl dissociates and exists in its protonated form as the C_6_H_5_NH_3_^+^. Therefore, the organic species ultimately incorporated into the perovskite structure is C_6_H_5_NH_3_^+^, which serves as the organic spacer cation. The resulting solution was initially heated to 120 °C to ensure complete dissolution and homogeneous mixing of the precursors, then naturally cooled to 50 °C to induce controlled nucleation via a mild temperature gradient [25,26].

**Figure 1:**
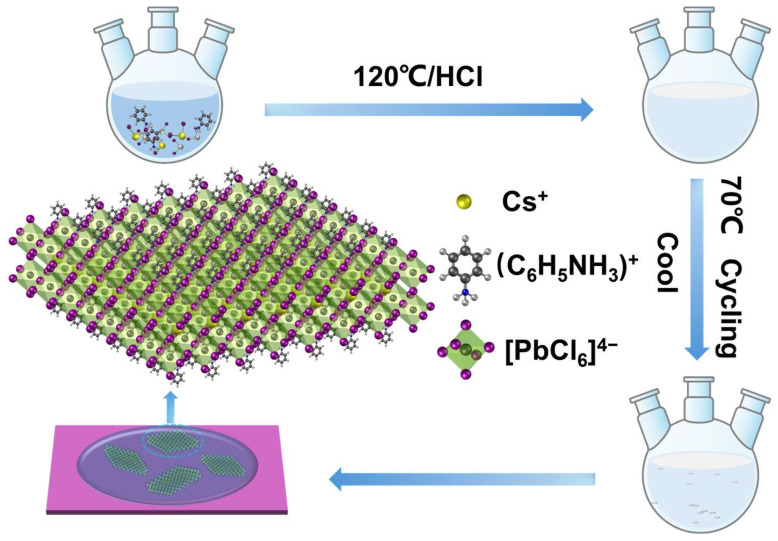
Schematic illustration of the synthesis procedure for (C_6_H_5_NH_3_)_2_CsPb_2_Cl_7_ perovskite.

Figure 1 illustrates the cooling crystallization process used in this study. The RP phase structure consists of alternating organic cation (C_6_H_5_NH_3_^+^) bilayers and inorganic [PbCl_6_]^4−^ octahedral bilayers, stacked in a typical RP phase perovskite layered arrangement. To further investigate the impact of annealing temperature on crystal size and crystallinity, optical microscopy was used to analyze the morphology of the perovskite synthesized at different annealing temperatures. The results show that under annealing conditions at 120 °C and 70 °C, the samples exhibit high uniformity and well-ordered morphology, further confirming the crucial role of crystallization dynamics in determining the final morphology of the material (Figure S1).

**Figure 2:**
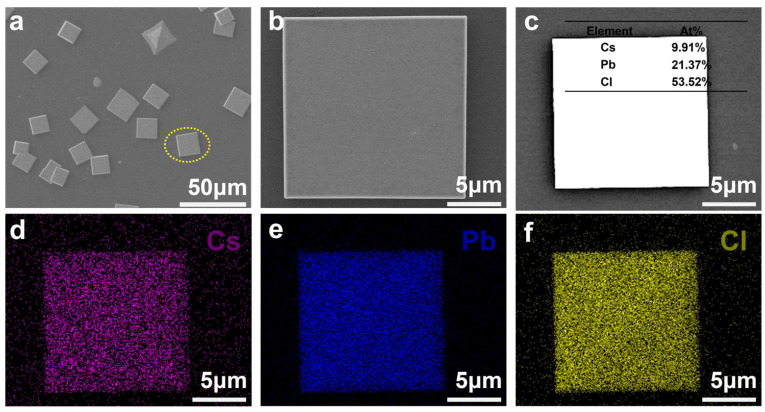
SEM morphology images and EDS elemental mapping of the (C_6_H_5_NH_3_)_2_CsPb_2_Cl_7_ microplates. (**a**) Low-magnification SEM image of the as-prepared sample. (**b**) High-magnification SEM image of the selected region from panel (**a**). (**c**) Corresponding EDS mapping overview; the inset shows the semi-quantitative EDS elemental proportion spectrum of the tested sample. (**d**–**f**) Elemental mapping images of Cs, Pb, and Cl, respectively.

To gain deeper insight into the microscopic morphological features, scanning electron microscopy (SEM) was employed for detailed characterization. As shown in Figure 2a, the as-prepared 2D perovskites exhibit relatively uniform lateral dimensions and well-defined, regular geometries, suggesting a controlled growth process. A representative square-shaped micron-sized plate was selected for further magnified observation (Figure 2b), which reveals a smooth and flat surface with a lateral size of approximately 20 μm. Such a flat and continuous morphology is beneficial for reducing surface irregularities and provides a suitable platform for subsequent optoelectronic measurements. In addition, to investigate the elemental composition and spatial distribution, a perovskite crystal was randomly selected for energy-dispersive X-ray spectroscopy (EDS) analysis. As shown in Figure 2c, cesium (Cs), lead (Pb), and chlorine (Cl) are clearly detected, with atomic percentages of 9.91%, 21.37%, and 53.52%, respectively. These experimentally obtained ratios are generally consistent with the expected stoichiometry of (C_6_H_5_NH_3_)_2_CsPb_2_Cl_7_, indicating that the main constituent elements are successfully incorporated. Moreover, Figure 2d–f shows that the main elements Cs, Pb, and Cl are uniformly distributed throughout the (C_6_H_5_NH_3_)_2_CsPb_2_Cl_7_ crystals. Complementary optical microscopy images (Figure S2) show well-defined square-shaped crystals with sharp edges, which is consistent with the regular morphology observed in SEM.

**Figure 3:**
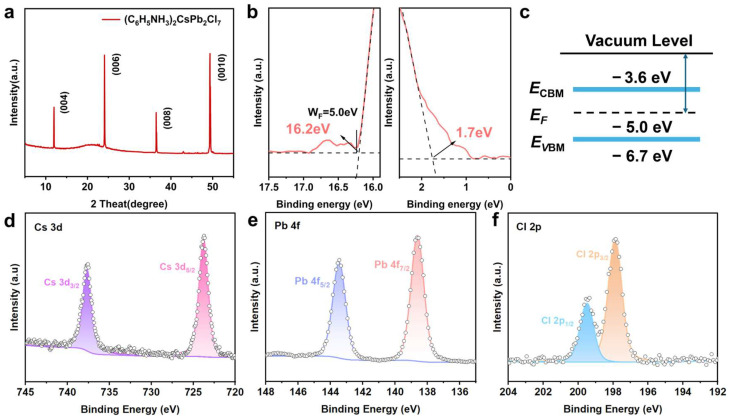
Structural and compositional analyses of the (C_6_H_5_NH_3_)_2_CsPb_2_Cl_7_ perovskite. (**a**) XRD pattern of the as-prepared sample. (**b**) UPS spectrum. (**c**) Electronic band structure diagram of the material. (**d**–**f**) High-resolution XPS spectra of Cs, Pb, and Cl elements, respectively.

Based on systematic morphological characterization, the crystal structure of (C_6_H_5_NH_3_)_2_CsPb_2_Cl_7_ was investigated using X-ray diffraction (XRD). The XRD patterns (Figure 3a) display a series of equally spaced (00l) diffraction peaks, which are consistent with the characteristic features of layered RP perovskites reported in the literature [2,27]. No diffraction peaks clearly attributable to impurity phases are observed. The exclusive presence of (00l) reflections indicates a strong preferred orientation along the c-axis and, together with the sharpness of the peaks, suggests a high degree of crystallinity and well-ordered stacking in the layered structure. When considered alongside the near-stoichiometric elemental ratios obtained from EDS measurements and their general agreement with previously reported n = 2 RP perovskites [28], these observations collectively suggest that the newly synthesized 2D layered RP perovskite is structurally consistent with an n = 2 RP phase. Although an exact structural assignment remains challenging in the absence of reference patterns.

**Figure 4:**
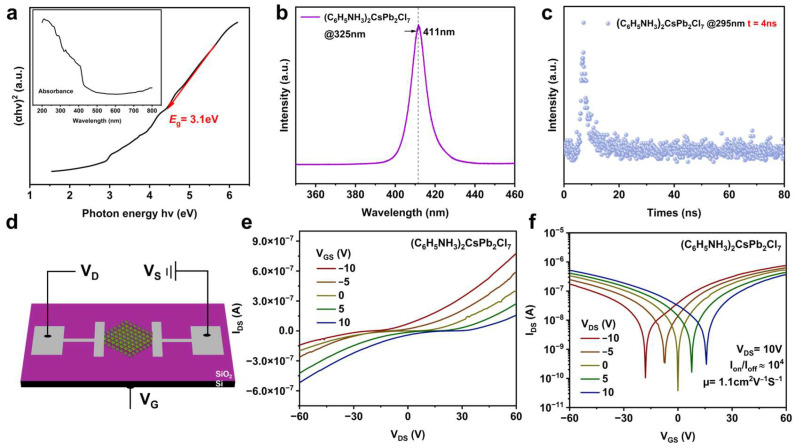
Optoelectronic and electrical characterizations of the layered (C_6_H_5_NH_3_)_2_CsPb_2_Cl_7_ RP perovskite and its corresponding device. (**a**) Tauc plot for bandgap calculation, with the inset showing the corresponding UV–Vis absorption spectrum. (**b**) Steady-state PL spectrum of (C_6_H_5_NH_3_)_2_CsPb_2_Cl_7_. (**c**) TRPL decay curve. (**d**) Schematic illustration of the electrode configuration of the FET device. (**e**) Output characteristic curves of the FET device. (**f**) Transfer characteristic curve of the FET device.

To systematically elucidate the electronic band structure of the 2D perovskite (C_6_H_5_NH_3_)_2_CsPb_2_Cl_7_, we combined UPS and UV–Vis absorption spectroscopy to comprehensively characterize and construct its complete band alignment. Specifically, the valence band maximum (VBM) was determined via UPS measurement, which was calculated to be −6.7 eV relative to the vacuum level (Figure 3b and Text S1). These UPS results provide direct experimental information on the occupied electronic states near the valence-band edge and establish the basis for constructing the band structure. The optical bandgap (*E*_g_) was estimated to be 3.1 eV from the Tauc plot derived from the UV–Vis absorption spectrum (Figure 4a and Text S1). Based on the measured VBM and optical bandgap, the conduction band minimum (CBM) was estimated to be −3.6 eV relative to the vacuum level, and the corresponding band structure of (C_6_H_5_NH_3_)_2_CsPb_2_Cl_7_ was constructed accordingly (Figure 3c). It should be noted that the CBM value was derived from the combination of UPS and optical measurements rather than being measured directly. In addition, the Fermi level (*E*_F_), as determined from the UPS measurement, is located at approximately −5.0 eV. Since the Fermi level lies within the optical bandgap and is closer to the CBM than to the VBM, the as synthesized crystals can be considered to exhibit weak n-type semiconducting characteristics. Such an assignment is consistent with the relative position of the Fermi level in a semiconductor energy level diagram.

The chemical states of the constituent elements in the as-prepared (C_6_H_5_NH_3_)_2_CsPb_2_Cl_7_ perovskite were examined by X-ray photoelectron spectroscopy (XPS). All spectra were calibrated by referencing the C 1s peak to 284.8 eV with charge correction applied using Thermo Scientific Avantage software (version 5.9931). Since carbon is intrinsically present in the organic component of the material, the C 1s signal may include contributions from both intrinsic carbon species and adventitious surface carbon. The spectra were fitted using a Shirley background and Gaussian-Lorentzian mixed functions, and the detailed fitting parameters, including peak positions, full width at half maximum, and fitting components, are summarized in Table S1.

As shown in Figure 3d, the high-resolution Cs 3d spectrum exhibits two peaks at 724.2 and 738.3 eV, corresponding to Cs 3d_5/2_ and Cs 3d_3/2_, respectively. The Pb 4f spectrum (Figure 3e) shows two peaks at 138.2 and 143.1 eV, which are assigned to Pb 4f_7/2_ and Pb 4f_5/2_, respectively, consistent with Pb^2+^ in the perovskite lattice. In the Cl 2p spectrum (Figure 3f), the peaks at 198.3 and 199.9 eV are attributed to Cl 2p_3_/_2_ and Cl 2p_1_/_2_, respectively. These results are consistent with the expected chemical states of the constituent elements in the layered perovskite. In addition, Fourier transform infrared spectroscopy (FTIR) characterized the organic functional groups within the material (Figure S3). The spectrum clearly displays characteristic absorption peaks attributable to the stretching vibrations of carbon-carbon double bonds (C=C) within the benzene ring skeleton, carbon–hydrogen bonds (C–H), and nitrogen–hydrogen bonds (N–H), confirming the stable presence of organic cations (C_6_H_5_NH_3_^+^) within the crystal structure.

We estimated the optical bandgap of (C_6_H_5_NH_3_)_2_CsPb_2_Cl_7_ based on the UV–Vis absorption spectrum using the Tauc plot method, where the plot of (αhν)^2^ versus photon energy (hν) was employed assuming a direct allowed transition. By linearly extrapolating the absorption edge, the bandgap was determined to be approximately 3.1 eV (Figure 4a and Text S1). To further analyze this result, we compared the electronic structure of (C_6_H_5_NH_3_)_2_CsPb_2_Cl_7_ with representative lead-free bismuth-based halides, such as Cs_3_Bi_2_I_9_. Previous studies have shown that Cs_3_Bi_2_I_9_ typically exhibits an indirect or mixed bandgap, often accompanied by very weak or even negligible photoluminescence and low quantum yield due to dominant non-radiative recombination processes [29]. In contrast, (C_6_H_5_NH_3_)_2_CsPb_2_Cl_7_ exhibits a sharp absorption edge, strong photoluminescence emission, and a relatively high photoluminescence quantum yield (29.8%), which is significantly higher than that of Cs_3_Bi_2_I_9_. These observations are more consistent with direct-like optical transition behavior than with strongly indirect optical behavior.

Steady-state photoluminescence (PL) spectra indicate that (C_6_H_5_NH_3_)_2_CsPb_2_Cl_7_ exhibits a violet emission peak at 411 nm with a single-peak feature and no evident side peaks. The full width at half maximum (FWHM) is approximately 10 nm, suggesting a concentrated emission channel with high color purity (Figure 4b). The PLQY is measured to be around 29.8% (Figure S4), which is considered moderate-to-high for an unoptimized 2D perovskite system, reflecting the intrinsic emissive potential of the material. TRPL decay curves display a biexponential behavior, with a fast component τ_1_ = 1.18 ns (amplitude B_1_ = 93.3) and a slow component τ_2_ = 5.9 ns (amplitude B_2_ = 23.9). The weighted average lifetime (τ_ave_), calculated according to the corresponding formula, is approximately 4 ns, indicating nanosecond-scale recombination dynamics, with no long-lived emission associated with deep traps observed (Figure 4c). Combining the TRPL and PLQY measurements, the radiative and non-radiative recombination rates were estimated to be K_r_ ≈ 7.45 × 10^7^ s^−1^ and K_nr_ ≈ 1.76 × 10^8^ s^−1^, respectively, suggesting that radiative recombination contributes significantly to the overall decay while non-radiative pathways remain non-negligible. Combining TRPL and PLQY data, it can be reasonably inferred that effective radiative recombination channels are established, although some non-radiative losses remain. Compared with previously reported unoptimized 2D RP perovskites (Table 1), (C_6_H_5_NH_3_)_2_CsPb_2_Cl_7_ demonstrates relatively favorable radiative recombination characteristics.

To systematically evaluate the charge transport properties of the 2D perovskite material (C_6_H_5_NH_3_)_2_CsPb_2_Cl_7_, field-effect transistor (FET) devices were fabricated using a bottom-gate, top-contact configuration with the perovskite serving as the active channel layer (Figure 4d). The output characteristics of the device, shown in Figure 4e, reveal a nearly linear increase in drain current with drain voltage in the low drain voltage region, indicating efficient carrier injection and suggesting good electrical contact between the perovskite channel and the source/drain electrodes. Moreover, the output characteristics measured at different gate voltages exhibit a continuous and tunable current response, indicating that the device possesses stable gate-voltage modulation capability.

Based on these results, we further investigated and analyzed the transfer characteristics of the device (Figure 4f). The device exhibits clear gate-voltage-dependent modulation, with an on/off current ratio of approximately 10^4^. From the transfer curve, the field-effect mobility was calculated to be 1.1 cm^2^ V^−1^ s^−1^ (Text S2). In addition, its electrical parameters were compared with those of previously reported related 2D perovskite devices (Table 2). To improve the validity of the comparison, the literature data were, as far as possible, restricted to systems without pronounced performance-enhancing strategies such as additional interface engineering, compositional doping, or device-structure optimization, so as to more appropriately reflect the intrinsic differences in device performance among the materials. The comparison indicates that the (C_6_H_5_NH_3_)_2_CsPb_2_Cl_7_ device delivers reasonably favorable mobility and switching characteristics, highlighting the potential of this material for charge transport and integrated device applications.

## 4. Conclusions

In conclusion, a novel chloride-based 2D RP perovskite, (C_6_H_5_NH_3_)_2_CsPb_2_Cl_7_, was successfully synthesized in this work, and its crystal structure and compositional characteristics, photophysical properties, and field-effect transport behavior were systematically investigated. The results show that this material possesses high crystallinity, a relatively well-ordered layered structure, and a smooth surface morphology, reflecting excellent structural order and crystal quality. Optical measurements show that it exhibits violet emission at 411 nm, with a full width at half maximum of only 10 nm and a bandgap of 3.1 eV, indicating a narrow emission bandwidth and high color purity. A PLQY of 29.8% and an average PL lifetime of 4 ns, together with the estimated recombination rates (K_r_ ≈ 7.45 × 10^7^ s^−1^; K_nr_ ≈ 1.76 × 10^8^ s^−1^), indicate appreciable radiative recombination accompanied by remaining non-radiative losses. FET based on this material achieved an on/off current ratio of approximately 10^4^ and a carrier mobility of 1.1 cm^2^ V ^−1^ s^−1^; compared with similar unoptimized 2D perovskite materials, these results indicate that it not only shows certain advantages in emission performance, but it also possesses appreciable in-plane charge transport capability. Overall, (C_6_H_5_NH_3_)_2_CsPb_2_Cl_7_ exhibits favorable radiative recombination characteristics and field-effect transport performance. This study highlights the potential of the chloride-based 2D RP perovskite (C_6_H_5_NH_3_)_2_CsPb_2_Cl_7_ for practical applications and provides a foundation for further optimization of its optoelectronic properties.

## Figures and Tables

**Table 1:** Comparison of optical properties of 2D perovskites without pronounced performance-enhancement strategies.

Perovskite	PL Peak (nm)	FWHM (nm)	PLQY (%)	τ_ave_ (ns)	K_r_ (10^7^ s^−1^)	K_nr_ (10^7^ s^−1^)	References
This work	411	11	29.8	4	7.45	17.6	\
(OAM)_2_CsPb_2_Cl_7_	433	12	1.85	1.1	1.68	89.2	[30]
(BZA)_2_PbCl_4_	450	\	3.57	3.96	9.02	24.4	[31]
(BZA)_2_PbBr_4_	415	\	4.98	0.71	7.01	13.4	[31]
(PEA)_2_PbBr_4_	483	20	~20	~5	4	16	[32]
(PEA)_2_PbCl_4_	580	260	2.2	26.7	0.08	3.7	[32]
(PMA)_2_PbBr_4_	424	\	7.35	\	\	\	[33]
(PMA)_2_Pb_2_Cl_4_	560	270	2.39	\	\	\	[33]
(BA)_2_PbCl_4_	595	292	0.63	\	\	\	[33]
TMPDAPbBr_4_	561	163	12.8	12	1.07	72.7	[34]

**Table 2:** Comparison of electrical performance of 2D perovskite FET without pronounced performance-enhancement strategies.

Perovskite	I_on_/I_off_	μ (cm^2^·V^−1^·s^−1^)	References
This work	10^4^	1.1	\
(BA)_2_MAPb_2_I_7_	10^6^	0.5	[35]
(BA)_2_(MA)_2_Pb_3_I_10_	10^6^	1.25	[36]
(PEA)_2_PbI_4_	/	1	[37]
(PEA)_2_PbCl_4_	10^2^	0.14	[35]
(PEA)_2_PbBr_4_	/	0.09	[35]
(PEA)_2_SnCl_4_	10^6^	3.6	[38]
(BA)_2_PbI_4_	10^3^	1.25	[39]
TEA_2_SnI_4_	10^4^	0.34	[40]
BDASnI_4_	10^4^	0.58	[9]
4Tm_2_SnI_4_	10^4^	2.32	[41]

## Data Availability

The original contributions presented in this study are included in the article/Supplementary Materials. Further inquiries can be directed to the corresponding author.

## Supplementary Materials

The following supporting information can be downloaded at: https://www.mdpi.com/article/10.3390/ma19101991/s1, Text S1. The band structure of the (C_6_H_5_NH_3_)_2_CsPb_2_Cl_7_ perovskite in this study was calculated by combining ultraviolet photoelectron spectroscopy (UPS) and ultraviolet absorption spectroscopy data; Text S2. Performance calculations for FETs; Text S3. Calculation of radiative and non-radiative recombination rates; Table S1. Fitting parameters of high-resolution X-ray photoelectron spectroscopy (XPS) spectra for (C_6_H_5_NH_3_)_2_CsPb_2_Cl_7_; Figure S1. Effect of annealing temperature on (C_6_H_5_NH_3_)_2_CsPb_2_Cl_7_ dimensions. (a–c) Correlation between initial annealing temperature and (C_6_H_5_NH_3_)_2_CsPb_2_Cl_7_ growth. Statistical analysis of two-dimensional perovskite dimensions grown at 115, 120 and 125°C. (d–f) Statistical distribution of perovskite growth dimensions under secondary annealing temperatures. Insert: Representative optical image of the two-dimensional perovskite (C_6_H_5_NH_3_)_2_CsPb_2_Cl_7_ synthesized under the corresponding conditions. Scale bar: 50 µm; Figure S2. Optical microscopy (OM) images of (C_6_H_5_NH_3_)_2_CsPb_2_Cl_7_; Figure S3. Fourier transform infrared (FTIR) spectrum of (C_6_H_5_NH_3_)_2_CsPb_2_Cl_7_; Figure S4. Photoluminescence quantum yield (PLQY) spectrum of (C_6_H_5_NH_3_)_2_CsPb_2_Cl_7_.
