# Supplementary material for: 2D Ruddlesden-Popper Perovskite (C6H5NH3)2CsPb2Cl7 with Favorable Radiative Recombination and Field-Effect Transport
Source: Materials (Basel). 2026 May 11;19(10):1991. doi: 10.3390/ma19101991 (PMC13209095; doi:10.3390/ma19101991)
Supplement: Supplementary file 1 [file materials-19-01991-s001.zip › materials-4302102-supplementary.pdf]

Supplementary materials

2D Ruddlesden-Popper Perovskite $(C_6H_5NH_3)_2CsPb_2Cl_7$ with Favorable Radiative Recombination and Field-Effect Transport

Zhe Pang[1], Yuxuan Wang [2](Co-first author), Chong Peng[1], Yingfei Liu[1], Jiaqian Que[1], Kefeiyang Hu[1], Xingbo Huang[1], and Yong Liu[1,*]

[1]School of Materials Science and Engineering, State Key Laboratory of Advanced Technology for Materials Synthesis and Processing, Wuhan University of Technology, Wuhan 430070, China; 15091422674@163.com (Z.P.); 15856589663@163.com (C.P.); liu714961@163.com (Y.L.); 2294599@whut.edu.cn (J.Q.); HKFY8789@whut.edu.cn (K.H.); 15251300417@163.com (X. H.)

[2]School of Mechanical and Electrical Engineering, Wuhan University of Technology, Wuhan 430070, China; 19870978239@163.com (Y.W)

* Correspondence author. Email: liuyong3873@whut.edu.cn;

Supplementary Text S1. The band structure of the $(C_6H_5NH_3)_2CsPb_2Cl_7$ perovskite in this study was calculated by combining ultraviolet photoelectron spectroscopy (UPS) and ultraviolet absorption spectroscopy data.

The energy position of the valence band maximum (E_{VBM}) was calculated from UPS data, using the formula $E_{VBM} = E_{cut} - E_{edge} - 21.22$ eV. Where 21.22 eV is the photon energy (hν) of the He I resonance line employed for UPS measurements; E_{cut} refers to the energy position of the secondary electron cutoff in the spectrum, which corresponds to the maximum kinetic energy signal of secondary electrons emitted from the material surface; E_{edge} is the energy value obtained by linear extrapolation of the steep rising portion of the valence band onset edge, defined as the intersection of the extrapolated linear line with the background noise baseline.

Determination of the conduction band minimum (E_{CBM}) requires optical bandgap data, which was acquired via the Tauc plot method from UV–Vis absorption spectroscopy. For semiconductor materials, electrons in both the valence and conduction bands are predominantly distributed in the vicinity of the forbidden bandgap. When the energy of incident photons is close to the bandgap, many electrons absorb the photon energy and undergo interband transitions, resulting in a marked increase in the absorption coefficient (α) with rising photon energy. The relationship between the optical bandgap (E_g) and the absorption coefficient (α) can be expressed as:

$$(\alpha h\nu)^{1/n} = A(h\nu - Eg) \quad \text{(S1)}$$

Where hν is the photon energy (1.99×10^{-25} J·m), A is a proportionality constant, and n is an exponent associated with the semiconductor type (n = 1/2 for direct bandgap semiconductors, while n = 2 for indirect bandgap semiconductors).

By plotting $(\alpha h\nu)^{1/n}$ on the y-axis and hν on the x-axis, the above relationship can be transformed into the linear equation: $y=C(x-E_g)$. After fitting the linear region of the obtained plot, the intersection of the fitted line with the x-axis gives the optical bandgap E_g.

Collectively, the optical bandgap E_g was measured using a UV–Vis spectrophotometer. Combined with the VBM energy (E_{VBM}) obtained from UPS characterization, the CBM energy (E_{CBM}) can be calculated via the equation: E_{CBM} =

$E_{VBM} + E_g$. On this basis, the complete energy band structure of the target material can be constructed.

Supplementary Text S2. Performance calculations for FETs.

The on/off current ratio (I_{on}/I_{off}), threshold voltage (V_{th}) and field-effect mobility (μ) were extracted from the field-effect transistor (FET) devices at a drain voltage (V_{DS}) of −10 V. The on/off ratio was determined from the transfer curves and defined as the ratio of the maximum on-state current to the minimum off-state current obtained from the transfer characteristic curves.

The field-effect mobility (μ) was calculated using the following equation:

$$\mu = \frac{I_{ds} \times L}{\mathrm{W}C_i \times \left(V_{gs} - V_{th}\right) \times V_{ds}} \tag{S2}$$

Where μ is the field-effect mobility, I_{DS} is the drain-source current, L and W are the channel length and channel width, respectively, C_i is the gate dielectric capacitance per unit area of the 300 nm thick silicon dioxide (SiO_2) layer (C_i = 1.1×10^{-8} F/cm^2), V_{GS} is the gate-source voltage (60 V), V_{th} is the threshold voltage, and V_{DS} is the drain-source voltage.

Supplementary Text S3. Calculation of radiative and non-radiative recombination rates.

The radiative recombination rate (k_r) and non-radiative recombination rate (k_{nr}) were estimated based on the measured photoluminescence quantum yield (PLQY) and time-resolved photoluminescence (TRPL) lifetime.

The PLQY is defined as:

$$\mathrm{P}LQY = \frac{K_r}{K_r + K_{nr}} \tag{S3}$$

The carrier lifetime (τ) obtained from TRPL measurements is given by:

$$\tau = \frac{1}{K_r + K_{nr}} \tag{S4}$$

where K_r is the radiative recombination rate, K_{nr} is the non-radiative recombination rate, PLQY is the photoluminescence quantum yield (expressed as a decimal), and τ is the average carrier lifetime obtained from TRPL measurements.

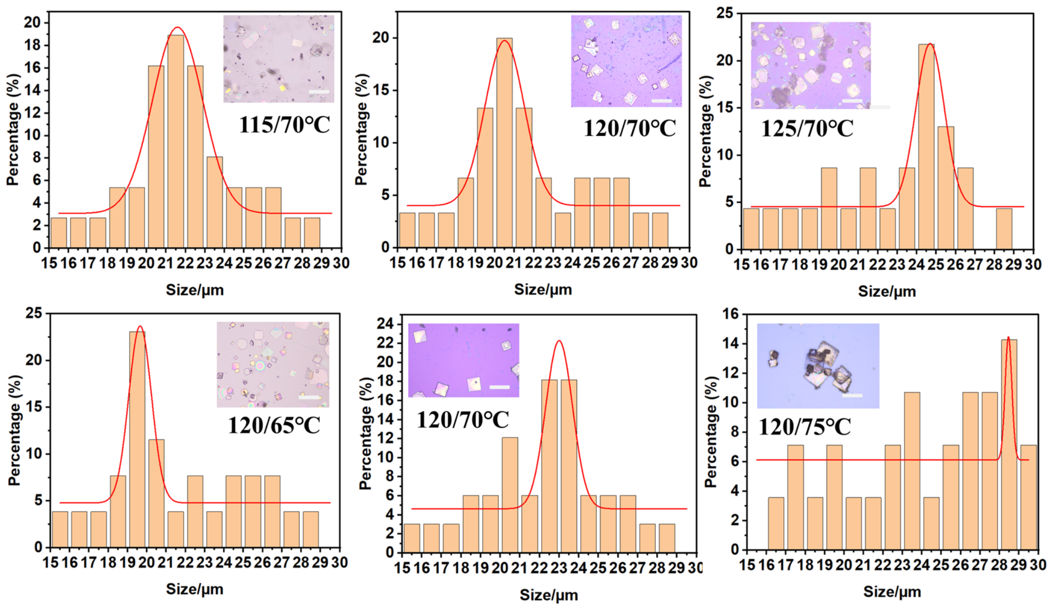

Figure S1. Effect of annealing temperature on $(C_6H_5NH_3)_2CsPb_2Cl_7$ dimensions. (a-c) Correlation between initial annealing temperature and $(C_6H_5NH_3)_2CsPb_2Cl_7$ growth. Statistical analysis of two-dimensional perovskite dimensions grown at 115, 120 and 125°C. (d-f) Statistical distribution of perovskite growth dimensions under secondary annealing temperatures. Insert: Representative optical image of the two-dimensional perovskite $(C_6H_5NH_3)_2CsPb_2Cl_7$ synthesized under the corresponding conditions. Scale bar: 50 μm.

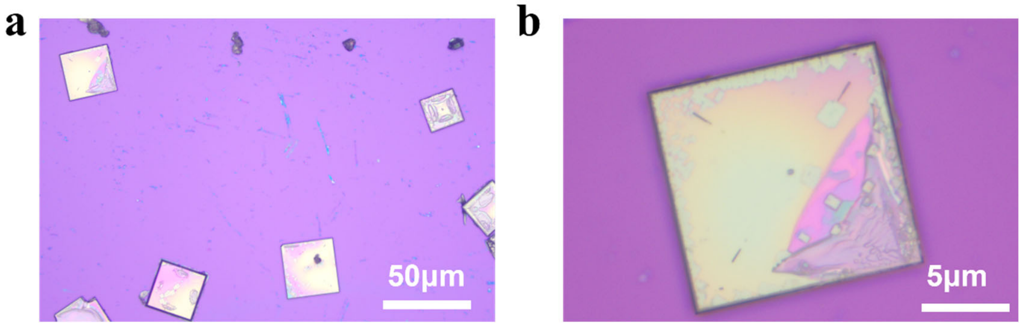

Figure S2. Optical microscopy (OM) images of $(C_6H_5NH_3)_2CsPb_2Cl_7$.

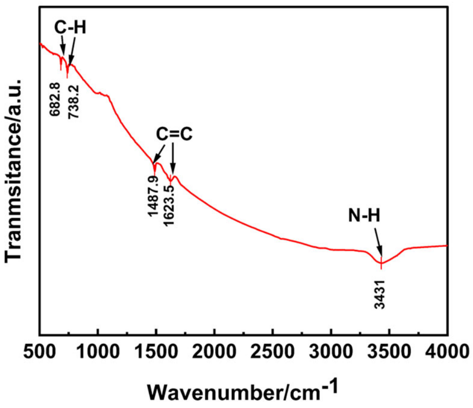

Figure S3. Fourier transform infrared (FTIR) spectrum of $(C_6H_5NH_3)_2CsPb_2Cl_7$.

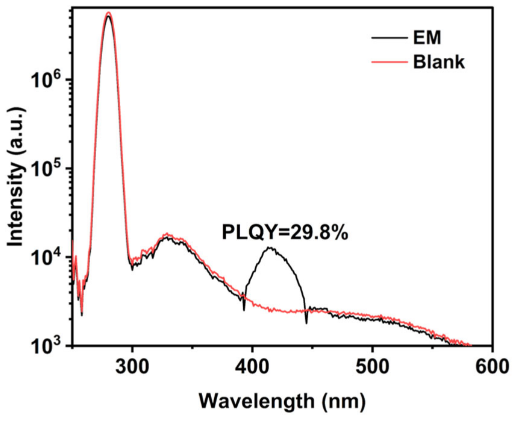

Figure S4. Photoluminescence quantum yield (PLQY) spectrum of $(C_6H_5NH_3)_2CsPb_2Cl_7$.

Table S1. Fitting parameters of high-resolution X-ray photoelectron spectroscopy (XPS) spectra for $(C_6H_5NH_3)_2CsPb_2Cl_7$

Fitting components	Position (eV)	FWHM (eV)	Area	Atomic%
C 1s	284.80	1.30	6432.01	56.54
Cs $3d_{5/2}$	723.63	1.30	19014.94	3.94
Cs $3d_{3/2}$	737.62	1.30	11432.73	2.39
Pb $4f_{7/2}$	138.65	0.95	19275.46	4.88
Pb $4f_{5/2}$	143.39	0.92	15380.47	3.90
Cl $2p_{3/2}$	197.80	0.96	5655.10	17.74
Cl $2p_{1/2}$	199.43	0.91	3384.64	10.62
